# Controllable assembly of silver nanoparticles induced by femtosecond laser direct writing

**DOI:** 10.1088/1468-6996/16/2/024805

**Published:** 2015-04-16

**Authors:** Huan Wang, Sen Liu, Yong-Lai Zhang, Jian-Nan Wang, Lei Wang, Hong Xia, Qi-Dai Chen, Hong Ding, Hong-Bo Sun

**Affiliations:** 1 State Key Laboratory on Integrated Optoelectronics, College of Electronic Science and Engineering, Jilin University, 2699 Qianjin Street, Changchun, 130012, People’s Republic of China; 2 College of Physics, Jilin University, 119 Jiefang Road, Changchun, 130023, People’s Republic of China; 3 State Key Laboratory of Inorganic Synthesis and Preparative Chemistry, College of Chemistry, Jilin University, 2699 Qianjin Street, Changchun, 130012, People’s Republic of China

**Keywords:** silver nanoparticles, femtosecond laser direct writing, patterning

## Abstract

We report controllable assembly of silver nanoparticles (Ag NPs) for patterning of silver microstructures. The assembly is induced by femtosecond laser direct writing (FsLDW). A tightly focused femtosecond laser beam is capable of trapping and driving Ag NPs to form desired micropatterns with a high resolution of ∼190 nm. Taking advantage of the ‘direct writing’ feature, three microelectrodes have been integrated with a microfluidic chip; two silver-based microdevices including a microheater and a catalytic reactor have been fabricated inside a microfluidic channel for chip functionalization. The FsLDW-induced programmable assembly of Ag NPs may open up a new way to the designable patterning of silver microstructures toward flexible fabrication and integration of functional devices.

## 1. Introduction

Recent advances in nanotechnology have resulted in rapid developments in nanomaterial synthesis methodologies [1–3]. Nanoparticles (NPs) with controllable size, shape, and polydispersity have been successfully prepared based on various material systems, ranging from inorganic carbon nanodots [4, 5] to organic nanocrystals [6, 7]. Among those zero-dimensional nanomaterials, metal nanoparticles (MNPs) that possess unique chemical/physical properties have sparked enormous research interest, revealing great potential toward a broad range of applications including electronics [8–10], sensors [11, 12], catalysis [13, 14], optics [15–20], and biomedicine [21]. Taking Ag NPs as a representative example, under certain excitation, localized surface plasmon resonance (LSPR) would occur among Ag NPs whose feature size is much smaller than the wavelength of the incident light. Consequently, Ag NPs could act as optical antennas to condense optical energy on their surface. Ag NPs have been considered a good candidate to fabricate surface-enhanced Raman scattering (SERS) substrates [22, 23]. Besides, as a non-toxic and stable inorganic material, Ag NPs have been used as fluorescent nanoclusters [24], electron acceptors [25, 26], catalysis active sites [27, 28], and a new generation of antimicrobials [29–32]. However, as compared with the refined synthetic routes for Ag NP preparation, from the practical point of view, there is a need for nanotechnologies that permit controllable assembly of these tiny NPs into desired micronanostructures for nanodevice fabrication. Nowhere is this more obvious than in some emerging fields such as plasmonic optical antennas, antibacterial, electrodes, and metasurfaces, where there is a need to organize NPs into rationally designed ensemble structures, even complex micropatterns.

This problem has motivated considerable efforts to develop improved patterning techniques toward nanodevice fabrication. For instance, electrostatic self-assembly [33–36], Langmuir–Blodgett technique [37–40], layer-by-layer assembly [41–45], and the use of solid templates [46–48] have been successfully developed for controllable Ag NP assemblies. Andrade *et al* reported a layer-by-layer assembly of Ag NPs on the surface of optical fiber tips for the development of remote SERS sensors [49]. The optical fiber tips were alternately soaked in 3-aminopropyltrimethoxysilane (APTMS) and Ag NPs several times to control the assembly. To get better control over the assembly process, solid templates could be used for patterning. Su *et al* fabricated Ag plasmonic waveguides by evaporation-induced self-assembly (EISA) of Ag NPs with the help of pillar array templates, which were fabricated beforehand through classical lithography [50]. In this regard, the patterning is limited to flat surfaces. Additionally, designable patterning of Ag NPs could also be achieved by direct printing of Ag NP inks [51–54]. Ahn *et al* fabricated Ag electrodes by moving the nozzle of Ag NP ink using a three-axis motion-controlled stage [55]. In this way, complex silver micropatterns could be directly printed. Nonetheless, the resolution is somewhat low; the narrowest line was about 2 *μ*m. Recently, direct femtosecond laser selective NP sintering (FLSNS) has been successfully developed to fabricate silver patterns [56]. Femtosecond laser irradiation would induce the sintering of Ag NPs; and the unirradiated region could be selectively removed, forming the desired silver micropatterns. This method could be considered as a subtraction-type fabrication; loading a uniform Ag NPs film is necessary for subsequent patterning. To date, despite there existing many successful examples that realized controllable assembly of Ag NPs, continued efforts are still desired in developing novel assembly strategies that permit flexible patterning of high-resolution silver micropatterns toward nanodevice fabrication.

In this work, we report a femtosecond laser direct writing (FsLDW)-induced controllable assembly of Ag NPs for making silver micropatterns. As a designable processing technique, FsLDW enables 3D fabrication, high-resolution prototyping, mask-free patterning, and flexible fabrication on non-planar substrates, revealing great potential in microdevice fabrication [57]. Herein, a focused femtosecond laser was used to drive and assemble Ag NP colloids into desired micropatterns. Taking advantage of the ‘direct writing’ feature, complex silver micropatterns such as Chinese knot, microelectrodes, and letters have been successfully fabricated without the use of any templates or masks. Moreover, the laser-induced assembly of Ag NPs enables flexible integration of silver microstructures with microdevices. As typical examples, we integrated a microheater inside a microfluidic chip for local heating and a catalytic reactor for H_2_O_2_ decomposition. The FsLDW-induced controllable assembly of Ag NPs may hold great promise for the fabrication and integration of metal NP-enabled nanodevices.

## 2. Experimental details

### 2.1. Preparation of Ag NP solution

Ag NPs were synthesized according to a reported method [58]. Typically, silver nitrate (1 mL, 0.1 mmol) and sodium malate (1 mL, 10 mmol) were added slowly into 97 mL of ultrapure water under vigorous stirring. After stirring for 10 min, sodium borohydride (1 mL, 10 mmol) was dropped into the preceding solution, followed by stirring for additional 2 min. To remove the residual salts in the solution, the obtained Ag NPs were purified by dialysis. After that, we obtained the Ag NPs aqueous solution for subsequent FsLDW.

### 2.2. FsLDW fabrication and integration of silver micropatterns

In a typical FsLDW fabrication, a tightly focused femtosecond laser beam with central wavelength of 800 nm and a pulse width of 120 fs was introduced into the interface between silver NPs solution and substrates. The tightly focused femtosecond laser would drive and trap the Ag NPs within the focal spot; in this way, assembled silver microstructures formed after sintering of Ag NPs along the traces of laser focus, which was guided by computer programs. In the integration experiment, microfluidic channels were fabricated by photopolymers (SU-8) through lithography. Gold pads for connecting Ag electrodes were deposited by vacuum thermal evaporation.

### 2.3. Characterization

The Ag NPs were characterized by transmission electron microscopy (TEM, JEM-2100F). Scanning electron microscopy (SEM, JSM7500), atomic force microscopy (AFM, Digital Instruments Nanoscope IIIA), and optical microscopy (OM, IBE2000) were used to observe the morphology of Ag assembly structures. Voltage applied on a microheater was offered by electrochemical workstation (CHI660E).

## 3. Results and discussion

**Figure 1.**
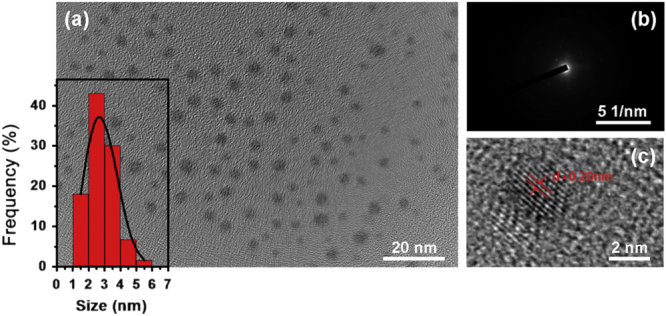
(a) TEM image of the as-obtained Ag NPs. The inset is the particle size distribution of the Ag NPs. (b) SAED pattern of the Ag NPs. (c) HRTEM image of a single Ag NP, (200) planes of the Ag NP with a d spacing of 0.2 nm could be observed.

In this work, malate-capped Ag NPs were used for the FsLDW fabrication. To evaluate the quality of the Ag NP suspension, the obtained Ag NPs were characterized by TEM. As shown in figure 1, the Ag NPs are uniform in size and their average diameter is 2.5 nm (inset of figure 1(a)). Figure 1(b) shows a selected area electron diffraction (SAED) pattern of the Ag NPs, indicating their crystalline structures. This was further confirmed by the high-resolution transmission electron microscopy (HRTEM) image (figure 1(c)), in which the (200) planes of Ag NPs with a spacing of 0.20 nm could be clearly observed.

**Figure 2.**
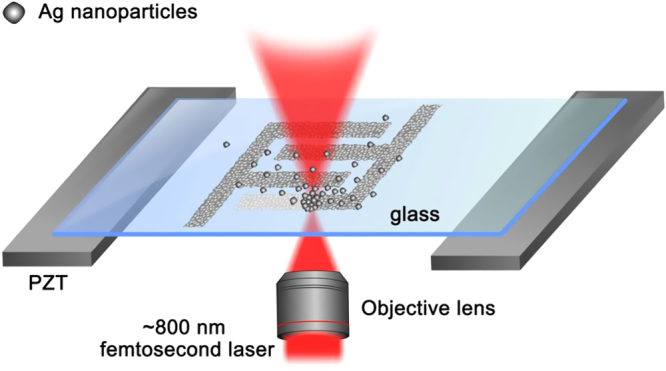
Schematic illustration of FsLDW-induced controllable assembly of Ag NPs. The laser beam was tightly focused by a ×100 oil immersion objective lens with a high numerical aperture NA = 1.45; the moving stage (PZT: piezoelectric transducer) was controlled by computers.

The obtained Ag NPs have been used for FsLDW directly without the use of any photosensitive reagents. Figure 2 shows the schematic illustration of the FsLDW of Ag NPs. When a tightly focused femtosecond laser beam irradiated into the Ag NP suspension, three radiation pressures including absorption force, scattering force, and gradient force were exerted on the tiny particles. Generally, absorption force and scattering force were proportional to laser intensity, which were applied in the direction of laser beam, whereas gradient force was proportional to the gradient of laser intensity, being toward the focus [59]. Although detailed theories to calculate these forces are still under development, it is very clear that the light–matter interaction is dominated by particle sizes, refractive index, and dielectric constant of target NPs [60–62]. In this experiment, considering the metallic Rayleigh particles (diameter *d* ≪ wavelength *λ*) are much smaller than the wavelength of laser (here *d* ≈ 2.5 nm; *λ* ≈ 800 nm), a gradation force proportional to the polarizability of an NP and the square of optical electric field vector dominates the assembly of Ag NPs, whereas absorption force and scattering force push the Ag NPs along the laser beam direction, leading to the enrichment of Ag NPs in the focal region. Together with the Ag NP deposition, mass transfer was performed by concentration diffusion of the Ag NPs solution. At the same time, due to the extremely high transient power density of the femtosecond laser, the aggregated Ag NPs have been sintered together. Since the near-infrared femtosecond laser beam is not absorbed by the solution, the beam penetrates deeply into the Ag NP solution with very small power loss. By scanning the laser focus along a preprogrammed trace, the desired silver micropatterns could be fabricated accordingly.

**Figure 3.**
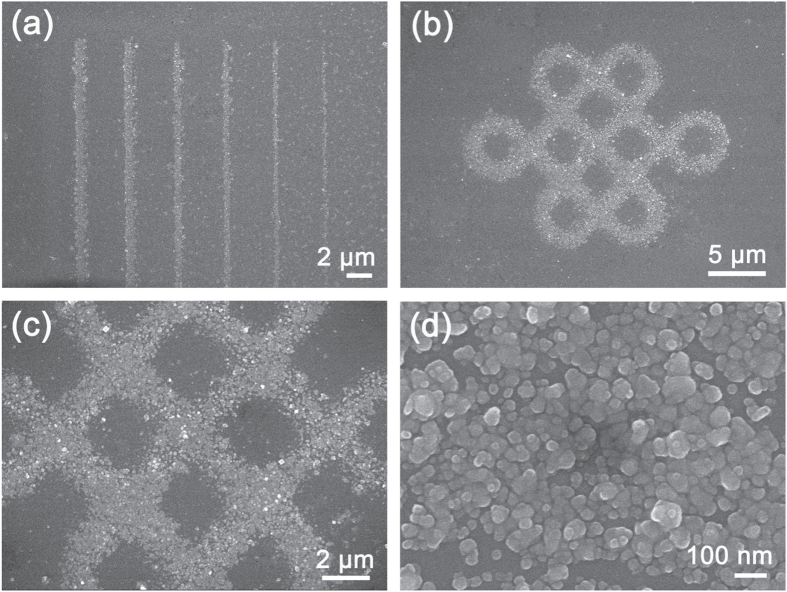
SEM images of silver micropatterns fabricated by FsLDW-induced controllable assembly of Ag NPs. (a) Ag microwires with different widths. From right to left, the width is 190, 290, 400, 510, 630, and 710 nm, respectively. (b) SEM image of a ‘Chinese knot’ pattern. (c), (d) Magnified SEM images of the ‘Chinese knot’ pattern.

Note that the pulse width of our femtosecond laser is only 120 fs, so thermal relaxation could be effectively suppressed within a small area near the focus. Taking advantage of this feature, high-resolution silver patterns could be fabricated. Figure 3 shows SEM images of the resulting silver micropatterns. The narrowest width of a continuous silver microwire was only ∼190 nm, which is much smaller than the light diffraction limit. The improved resolution could be mainly attributed to the use of an ultrafast laser, which induces nonlinear effects such as multiphoton absorption [63]. In addition to simple microwires, any desired micropatterns could be readily fabricated through FsLDW. As an example, we fabricated a ‘Chinese knot’ structure on a glass substrate (figure 3(b)). A magnified SEM image (figure 3(c)) showed that the line width of the ‘Chinese knot’ was ∼2 *μ*m. By further magnifying the SEM images, we found that the silver micropattern was constructed by close-packed silver particles whose size grew to tens of nanometers. As compared with the TEM image of the pristine Ag NPs, whose size is below 5 nm, the significant increase in particle size in the silver micropattern could be attributed to the laser-induced sintering.

**Figure 4.**
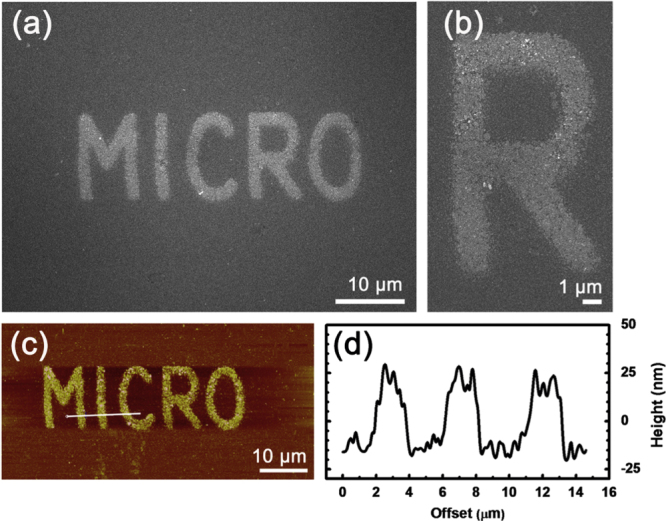
(a) SEM image of a silver word (MICRO) fabricated by FsLDW-induced controllable assembly of Ag NPs. (b) SEM image of the letter R. (c) AFM image of the silver word (MICRO). (d) Height profile measured along the white line shown in (c).

To illustrate its full potential in flexible patterning, a word (MICRO) was successfully fabricated through FsLDW of Ag NPs. Figures 4(a) and (b) show SEM images of the MICRO pattern and the letter ‘R’, respectively. The average line width of these letters is measured to be ∼2 *μ*m. To get further insight into the surface morphology of these letters, they were characterized by AFM. As shown in figure 4(c), MICRO could be clearly indentified from the image, in good agreement with the SEM image (figure 4(a)). The profile of the micropattern was also measured along a white line in figure 4(c). The micropattern is about 50 nm in thickness. Notably, the surface of the micropattern is very rough; its surface roughness (Rq) is measured to be about 11 nm. The rough surface could be attributed to the close packing of silver particles. Considering the rough surface, silver electrodes fabricated using this method may find some applications in electroanalysis.

**Figure 5.**
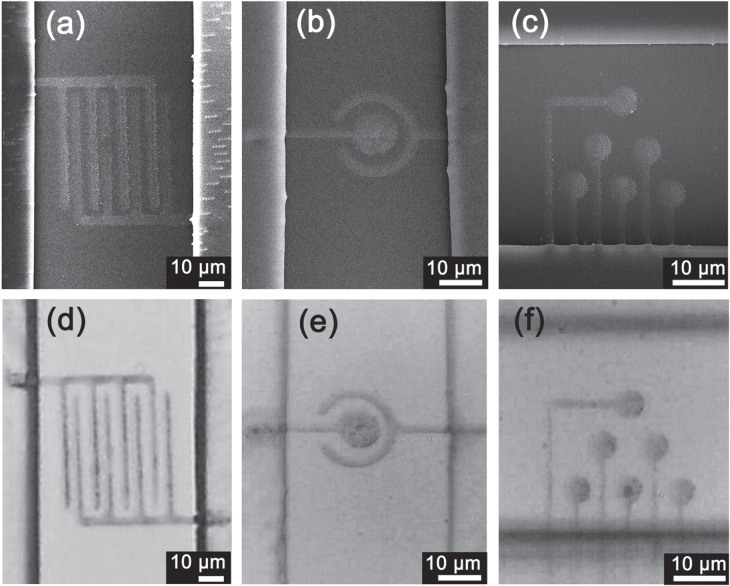
SEM images (a)–(c) and corresponding optical images (d)–(f) of three kinds of silver microelectrodes integrated with a microfluidic device. (a), (d) Interdigital electrode, (b), (e) two-electrode system, and (c), (f) MEA.

To demonstrate the capability of integrating such silver micropatterns with given microdevices, three kinds of microelectrodes were successfully integrated within a microfluidic chip by FsLDW-induced controllable assembly of Ag NPs (figure 5). As typical examples, a pair of interdigital electrodes, and a ring-circle working-counter electrode have been patterned. Note that both the two electrodes are widely used in electroanalysis for current/impedance detection; FsLDW-induced controllable assembly of Ag NPs may find broad application in highly sensitive electroanalysis, especially for bioelectricity signal collection. In this case, microelectrode arrays (MEAs), whose sizes were similar to cells, have been fabricated, as shown in figures 5(c) and (f). Independent electrodes in MEAs could be utilized to collect potential signals transferring in organisms, or collect signals at different points from the same cells.

**Figure 6.**
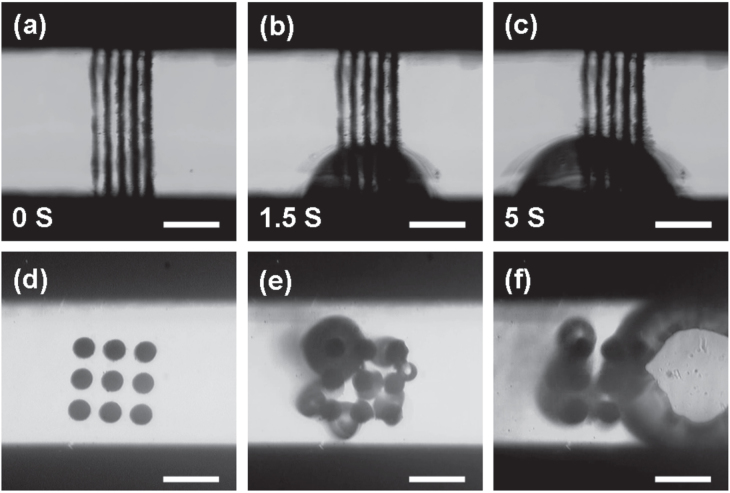
Silver microheater and catalytic microreactor fabricated by FsLDW-induced controllable assembly of Ag NPs. (a)–(c) Heating process of a microheater integrated within a microfluidic channel. A microbubble could be clearly observed, indicating the heating of the solvent. (d)–(f) Catalytic decomposition of H_2_O_2_ inside a silver microreactor. (e) Gas bubbles appeared as soon as H_2_O_2_ was injected into the channel; (f) 2 s later, the bubble grew bigger. The scale bar is 20 *μ*m.

For practical applications, a silver microheater was fabricated by FsLDW-induced controllable assembly of Ag NPs (figure 6). Five parallel microwires were fabricated between two gold electrodes. The patterned silver structure is conductive (see supporting information figure S1), when a voltage of 2 V was supplied on the microheater, a bubble rose from the water within 1.5 s, indicating the high heating efficiency. With continuous heating, the gas bubble grew larger, indicating the increase of local temperature. Compared with general microheaters fabricated by conventional craft, FsLDW fabrication was compatible with many substrates ranging from inorganic glass and silicon to organic polymers such as polydimethylsiloxane (PDMS) and polymethyl methacrylate (PMMA). In this regard, the technique would be potentially important to multifunction integration of microfluidic chips. FsLDW-induced controllable assembly of Ag NPs enables flexible patterning of silver at any desired place. In addition to the microheater, silver microstrucutures could also be integrated with microfluidic devices for catalysis and pump applications. As shown in figures 6(d)–(f), we explored Ag-catalyzed decomposition of H_2_O_2_ inside a microfluidic channel; the generation of O_2_ could be observed as soon as H_2_O_2_ was injected into the microchannel. Since the Ag catalysts could be flexibly placed at any desired position, the generated oxygen bubble could be potentially used as a pump for directional transport of target objects or to guide the flow inside a microfluidic device [64].

In conclusion, FsLDW-induced controllable assembly of Ag NPs has been successfully developed for designable patterning of silver microstructures. High-resolution silver micropatterns with the high resolution of 190 nm have been readily fabricated according to preprogrammed models. SEM and AFM images proved that silver micropatterns were constructed by close-packed Ag particles with an average size of tens of nanometers, which indicates the sintering of Ag NPs during laser irradiation. As typical models, three microelectrode patterns have been fabricated within a microfluidic device, revealing its strong capability for post-integration. Additionally, a silver microheater and a silver-catalyst array have been successfully fabricated for localized heating and catalytic decomposition of H_2_O_2_, respectively. As a laser-mediated assembly route, FsLDW-induced controllable assembly of Ag NPs may find broad application in the fabrication of silver-based microdevices. Especially, for microfluidic devices, FsLDW-induced controllable assembly of Ag NPs may hold great promise for flexible integration of functional devices such as SERS substrates, conductive microelectrodes, microheaters, Ag catalysts, and pumps, within a microfluidic chip, contributing to the development of multifunctional microfluidic chips.
